# Early antiretroviral treatment (eART) limits viral diversity over time in a long-term HIV viral suppressed perinatally infected child

**DOI:** 10.1186/s12879-016-2092-z

**Published:** 2016-12-09

**Authors:** Paolo Palma, Paola Zangari, Claudia Alteri, Hyppolite K. Tchidjou, Emma Concetta Manno, Giuseppina Liuzzi, Carlo Federico Perno, Paolo Rossi, Ada Bertoli, Stefania Bernardi

**Affiliations:** 1 Academic Department of Pediatrics, Unit of Immune and Infectious Diseases, Children’s Hospital Bambino Gesù, P.zza Sant’Onofrio, 4-00165 Rome, Italy; 2 Research Unit in Congenital and Perinatal Infections, Children’s Hospital Bambino Gesù, Rome, Italy; 3 Department of Experimental Medicine and Surgery, Tor Vergata University, Rome, Italy; 4 Clinical Department, National Institute for Infectious Diseases ’L. Spallanzani’, Rome, Italy

**Keywords:** HIV, Early antiretroviral treatment, Children, Viral evolution, Immunotherapy

## Abstract

**Background:**

HIV genetic diversity implicates major challenges for the control of viral infection by the immune system and for the identification of an effective immunotherapeutic strategy. With the present case report we underline as HIV evolution could be effectively halted by early antiretroviral treatment (eART). Few cases supported this evidence due to the difficulty of performing amplification and sequencing analysis in long-term viral suppressed patients. Here, we reported the case of limited HIV-1 viral evolution over time in a successful early treated child.

**Case presentation:**

A perinatally HIV-1 infected infant was treated within 7 weeks of age with zidovudine, lamivudine, nevirapine and lopinavir/ritonavir. At antiretroviral treatment (ART) initiation HIV-1 viral load (VL) and CD4 percentage were >500,000 copies/ml and 35%, respectively. Plasma genotypic resistance test showed a wild-type virus. The child reached VL undetectability after 33 weeks of combination antiretroviral therapy (cART) since he maintained a stable VL <40copies/ml. After 116 weeks on ART we were able to perform amplification and sequencing assay on the plasma virus. At this time VL was <40 copies/ml and CD4 percentage was 40%. Again the genotypic resistance test revealed a wild-type virus. The phylogenetic analysis performed on the HIV-1 pol sequences of the mother and the child revealed that sequences clustered with C subtype reference strains and formed a monophyletic cluster distinct from the other C sequences included in the analysis (bootstrap value >90%). Any major evolutionary divergence was detected.

**Conclusions:**

eART limits the viral evolution avoiding the emergence of new viral variants. This result may have important implications in host immune control and may sustain the challenge search of new personalized immunotherapeutic approaches to achieve a prolonged viral remission.

**Electronic supplementary material:**

The online version of this article (doi:10.1186/s12879-016-2092-z) contains supplementary material, which is available to authorized users.

## Background

HIV-1 infection is characterized by broad genetic diversity and rapid evolution that influence the pathogenesis, transmission and clinical management of the infection [1]. Such high genetic variability represents also a major drawback in the identification of an effective immunotherapeutic strategy. Genetically homogeneous virus population has been found at birth in perinatally HIV infected infants, in contrast to the heterogeneous virus populations often found in their infected mothers [2]. Similarly, a uniform viral population has been observed in newly infected adults short after transmission [3]. These observations led to the hypothesis that an early intervention with cART could limit viral evolution. Indeed, the homogeneity of viral sequences in HIV infected individuals treated during early infection compared with higher diversity in late treated patients has been recently confirmed in adult populations [4–6]. Although the relationship between the viral evolutionary dynamics and timing of treatment has been explored to some extent in adult less is known about this relationship during paediatric HIV-1 infection [7]. This is mostly due to the difficulty of performing amplification and sequencing analysis with limited volume of blood in perinatally HIV infected children on viral suppression since infancy [7–9]. Here, we reported the case of limited HIV-1 viral evolution in an early treated child with stable viral VL <40 copies/ml in which we were able to amplify and analyze HIV-1 pol sequences at different time points.

## Case report

An infant was born by elective caesarean section, two hours after membrane rupture at 38 weeks of gestation to an HIV-1 infected woman who started a first line ART with lamivudine, zidovudine and lopinavir/ritonavir 1 week before the delivery. Maternal VL at delivery was 203,686 copies/ml. Intrapartum antiretroviral prophylaxis with intravenous zidovudine was administered and the child started postnatal prophylaxis with zidovudine at 6 h of birth for 6 weeks. Breastfeeding was avoided. Polymerase chain reaction (PCR) for genes GAG, POL, and ENV performed at 6 weeks of life was positive and his HIV-RNA VL was >500,000 copies/mL with CD4 lymphocyte percentage 35%.

The genotypic resistance test from both mother and child didn’t show any transmitted drug resistance for PI, NRTI or NNRTI drug classes. Based on these results, at 7 weeks of age cART was initiated. A four-drug regimen of zidovudine, lamivudine, nevirapine and lopinavir/ritonavir was selected according to the results of genotypic resistance test. Plasma VL remained detectable till the 32^th^ week from starting therapy despite an adequate maternal compliance with infant’s drugs and on subsequent medical check the baby maintained undetectable HIV-1 RNA and CD4 T count within the range for age. At 48 weeks from starting therapy, ART was simplified by suspending protease inhibitor. Concurrently to the VL undetectability, HIV-1 antibodies were negative in the child at 26, and 31 months of age. After 116 weeks on cART, we were able to perform viral isolation and amplification. At this time VL was stable <40 copies/ml (ABBOTT) and CD4 percentage was 40%. A genotypic resistance test for *pol* was re-performed and no-drug resistance was found for a second time.

In order to clarify the epidemiological linkage and the evolutionary divergence between mother and child HIV-1 strains, a phylogenetic analysis was carried out on pol sequences performed at different time points. In particular, one pol sequence from plasma HIV-RNA and one pol sequence from PBMCs HIV-1 DNA obtained at the time of partum and 2 years later, respectively, were available for the mother. Two plasma *pol* sequences at the time of birth and 2 years later (corresponding to the 116 week of ART), were available for the child. To define the HIV-1 subtype and the sequence inter-relationships between the mother/child pair a neighbor joining (NJ) tree [10] was constructed using a first dataset containing all *pol* sequences obtained from the mother and child, HIV pol reference sequences, and 396 full-length pol sequences (1,200 bp) obtained from routine laboratory testing at the Virology Unit Hospital “Tor Vergata”, from January 2012 to December 2014. The reliability of the branching orders was assessed by bootstrap analysis of 1000 replicates. Genetic distances were calculated using MEGA 6.0 based on Kimura-2 parameter (K2P) model [11]. To avoid potential contaminations identical sequences amplified in the same run were excluded. Phylogenetic analysis by NJ method revealed that the HIV-1 *po*l sequences from the mother/child pair clustered together with C subtype reference strains, forming a monophyletic cluster distinct from the other C sequences included in the analysis (bootstrap value >90%) (Additional file 1: Figure S1).

**Fig. 1:**
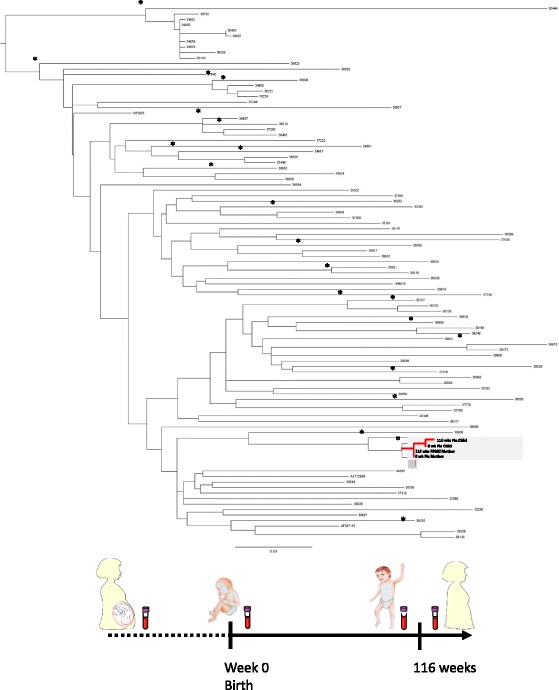
Maximum likelihood phylogenetic tree constructed on the pol gene sequences of 102 isolates and additional 4 subtype C references. The tree was rooted using the midpoint rooting method. Branch lenghts were estimated with the best fitting nucleotide substitution model (GTR + G + I) according to a hierarchical likelihood ratio test. The bar at the bottom indicating 0.03 nucleotide substitution per site. The astericks (*) along a branch represent bootstrap support >85%. The 4 C sequences belonged to mother to child transmission chain are shown in red and the cluster involving them is in the gray box. The cartoon summarizes the timing of sampling

Once the HIV-1 subtype was assigned, the statistical robustness of the monophyletic clade was confirmed also by the ML tree, containing only C reference sequences and 98 C isolates obtained from routine laboratory testing (bootstrap value >85%) (Fig. 1). This was inferred by the PhyML program (http://www.atgc-montpellier.fr/phyml/) using the GTR + I + Γ nucleotide substitution model. The simplest model that adequately fitted the sequence data was selected according to the Akaike Information Criterion (AIC) included in the MEGA package (version 6.0). Robustness of the phylogenetic clades was evaluated by bootstrap analysis (1000 replicates). The tree was rooted using a midpoint rooting. Again, the 4 pol sequences from the mother/child pair form a monophyletic cluster distinct from all the other C sequences included in the analysis (Fig. 1).

The extremely low mean genetic distance in pol region characterizing pol sequences from the mother/child pair compared to the ones from local unrelated non-cluster C controls (pol: 0.0017, standard error [SE]:±0.001 vs 0.081, standard error [SE]:±0.004) confirmed the high homology among mother and child sequences.

The phylogenetic analysis also revealed that the 2 sequences of the child clustered together and showed a minimal evolutionary divergence among them (mean ± SE:0.000090 ± 000087). This minimal evolutionary divergence is sustained by a single nucleotide substitution at position 231 of RT (C to T [F77F]).

## Conclusions

HIV-1 populations in the blood of the newly infected individuals are largely homogenous and evolve in a manner consistent with exponential viral replication [3]. Thus, starting antiretroviral treatment during acute infection can limit HIV viral evolution avoiding the emergence of new viral variants. Recent studies in HIV infected adults support this evidence [4–6] but few data are available in the pediatric setting [7]. The present case report highlights the impact of eART in limiting HIV genetic diversity over time in a perinatally HIV infected child on stable viral suppression (<40 copies/ml). Our result confirming those recently published in adults [4–6], may have important implications for the future defining of a personalized immunotherapeutic approach [12]. To date, eART alone is not sufficient to induce a sustained viral remission and additional immunotherapeutic interventions should be considered [12]. An effective early cART can prevent HIV-1 evolution modifying the natural history of the infection from a rapidly evolving viral infection to a state of clonal persistence with a single or a few variants. This restricted pool of variants can be more easily targeted by autologous cytotoxic T-lymphocytes (CTL) [13] or therapeutic vaccines induced strategies [12, 14]. Further studies are needed in order to determine whether limited HIV-1 evolution overtime can be associated with a higher likelihood to achieve viral remission [9].

## Additional files

Additional file 1: Figure S1. Neighborg phylogenetic tree constructed on the pol gene sequences of 400 isolates and additional 163 HIV-1 subtype references. The bar at the bottom indicating 0.01 nucleotide substitution per site. Bootstrap support >90% were showed along the branches. Isolates of sutypes C are shown in red. The sequences involved in mother to child transmission chain are in bold red. (PPTX 160 kb).

## Abbreviations

ART: Antiretroviral treatment
cART: Combination antiretroviral therapy
CTL: Cytotoxic T-lymphocytes
eART: Early antiretroviral treatment
NJ: Neighbor joining
PCR: Polymerase chain reaction
VL: Viral load
