# Supplementary figures and images for: Early antiretroviral treatment (eART) limits viral diversity over time in a long-term HIV viral suppressed perinatally infected child
Source: BMC Infect Dis. 2016 Dec 9;16:742. doi: 10.1186/s12879-016-2092-z (PMC5148894; doi:10.1186/s12879-016-2092-z)

## Slide 1
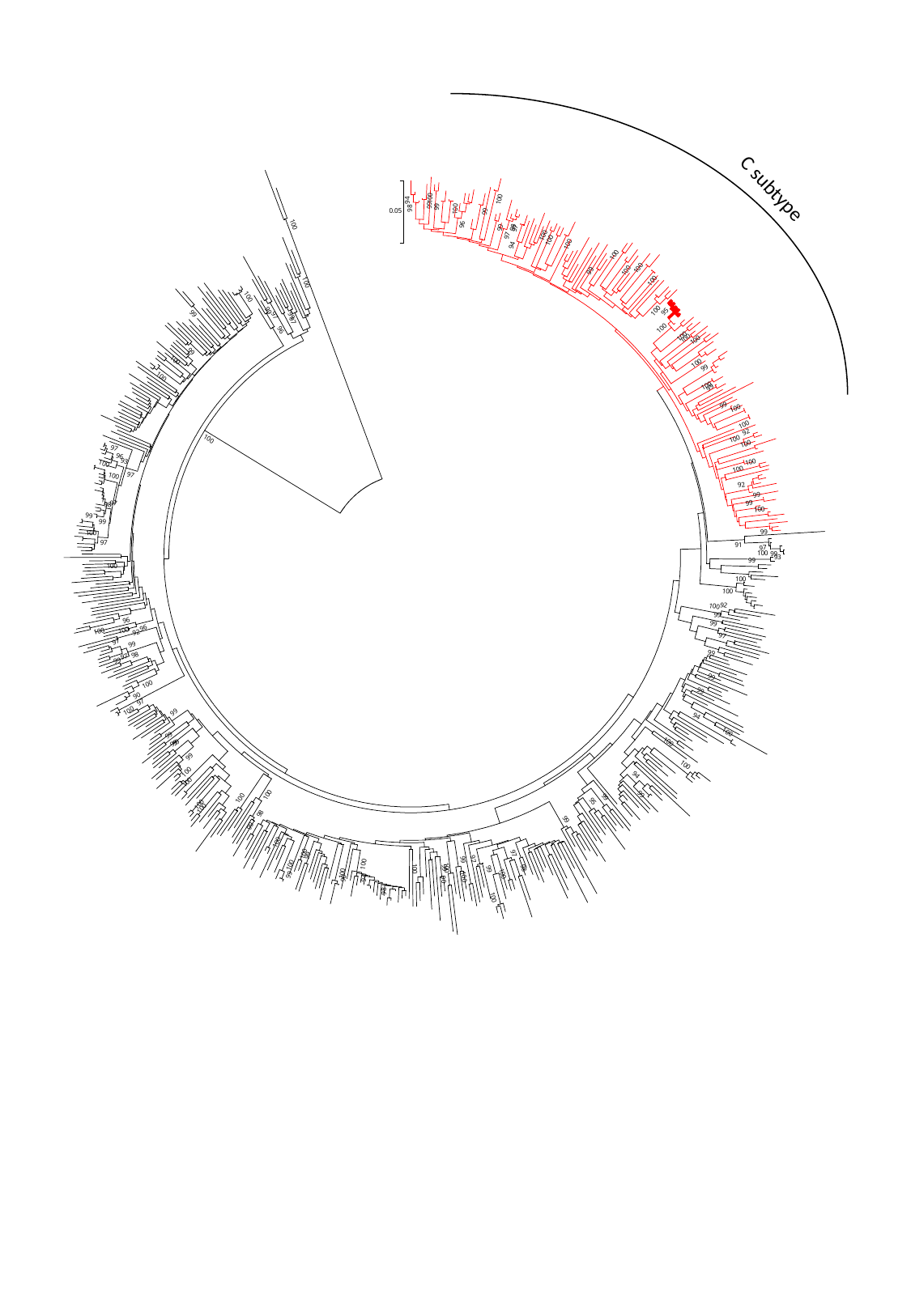

C subtype

Supplement: Additional file 1: Figure S1. — Neighborg phylogenetic tree constructed on the pol gene sequences of 400 isolates and additional 163 HIV-1 subtype references. The bar at the bottom indicating 0.01 nucleotide substitution per site. Bootstrap support >90% were showed along the branches. Isolates of sutypes C are shown in red. The sequences involved in mother to child transmission chain are in bold red. (PPTX 160 kb). [file 12879_2016_2092_MOESM1_ESM.pptx]
